# Detection of SARS-CoV-2 in Cancellous Bone of Patients with COVID-19 Disease Undergoing Orthopedic Surgery: Laboratory Findings and Clinical Applications

**DOI:** 10.3390/ijerph191710621

**Published:** 2022-08-25

**Authors:** Edoardo Guazzoni, Alberto Castelli, Alberto Polizzi, Giacomo Galanzino, Antonio Piralla, Federica Giardina, Fausto Baldanti, Eugenio Jannelli, Laura Caliogna, Gianluigi Pasta, Mario Mosconi, Federico Alberto Grassi

**Affiliations:** 1 Department of Orthopaedics and Traumatology, IRCCS Fondazione Policlinico San Matteo, University of Pavia, 27100 Pavia, Italy; 2 Microbiology and Virology Department, IRCCS Fondazione Policlinico San Matteo, 27100 Pavia, Italy; 3 Department of Clinical, Surgical, Diagnostic and Pediatric Sciences, University of Pavia, 27100 Pavia, Italy; 4 Multidisciplinary Department of Medico-Surgical and Dentistry Specialties, Luigi Vanvitelli, University of Campania, 80138 Naples, Italy

**Keywords:** COVID-19, bone, orthopedic surgery, operative room, risk of infection, tissue bank

## Abstract

An emerging issue for orthopedic surgeons is how to manage patients with active or previous COVID-19 disease, avoiding any major risks for the surgeons and the O.R. personnel. This monocentric prospective observational study aims to assess the prevalence of SARS-CoV-2 viral RT-PCR RNA in cancellous bone samples in patients with active or previous COVID-19 disease. We collected data about 30 consecutive patients from our institution from January 2021 to March 2021 with active or previous COVID-19 disease. The presence of SARS-CoV-2 in the samples was determined using two different PCR-based assays. Eighteen of the thirty patients included in the study had a positive nasopharyngeal swab at the time of surgery. Twelve patients had a negative nasopharyngeal swab with a mean days since negativization of 138 ± 104 days, ranging from 23 to 331 days. Mean days of positivity to the nasal swab were 17 ± 17. Twenty-nine out of thirty (96.7%) samples were negative for the presence of SARS-CoV-2 RNA. In one sample, low SARS-CoV-2 load (Cycle threshold (Ct) 36.6.) was detected but not confirmed using an additional confirmatory assay. The conducted study demonstrates the absence of the viral genome within the analyzed cancellous bone. We think that the use of personal protection equipment (PPE) to only protect from aerosol produced during surgery, both in active and recovered patients, is not strictly necessary. We think that the use of PPE should not be employed by surgeons and the O.R. personnel to protect themselves from aerosols produced from the respiratory tract. Moreover, we think that our results could represent a valid basis for further studies related to the possibility of bone donation in patients that suffered and recovered from COVID-19.

## 1. Introduction

On 11 March 2020, the World Health Organization (WHO) declared the SARS-CoV-2 disease as a global pandemic. Little is known about how SARS-CoV-2 infection can negatively affect the musculoskeletal system [1]. Otherwise, recent studies have shown that patients with moderate and severe coronavirus disease (COVID-19) have a variable involvement of the musculoskeletal system [2].

SARS-CoV-2 belongs to the coronavirus family and represents a new viral strain never identified in humans before. This virus is transmitted by air through the emission of droplets. Infection is favored by close contact among people, particularly in closed environments such as hospitals; therefore, nosocomial infections from SARS-CoV-2 are widely reported in the literature [3,4]. SARS-CoV-2 is thought to predominantly infect type-II pneumocytes that line the respiratory epithelium, which express ACE2 and TMPRSS2. Disser et al. have shown that in the musculoskeletal system many cells express TMPRSS2 (pericytes, muscle stem cells, macrophages, adaptive immune cells (B, T, or natural killer cells), and myonuclei (muscle fibers), though only pericytes and smooth muscle cells express ACE2 [2].

However, it is important to emphasize that air transport may not be the only way of transmission. In particular, contagion by direct or indirect contact with mucous membranes of the eyes, mouth, and nose has been described [5]. SARS-CoV-2 is responsible for respiratory and flu-like symptoms. In more severe cases, the infection may cause pneumonia, acute respiratory distress syndrome, and onset of acidosis with kidney damage. Death may occur due to the progression of these conditions. Furthermore, recent studies have shown an increased thrombotic risk in patients with COVID-19 disease [6].

The COVID-19 outbreak resulted in a severe reduction in operating capacity all around the world and cessation of routine elective surgery to reduce the pressure on the intensive care unit (ICU) [3,5].

Due to the COVID-19 pandemic, resources have been allocated to ICUs and COVID-19 wards and many “non-urgent” orthopedic surgeries have been postponed or canceled to preserve medical resources. The “Non urgent orthopaedic surgery” was defined as planned non-trauma-related surgery [7,8,9].

An emerging issue for orthopedic surgeons is how to manage patients with active COVID-19 disease or patients who have recovered from it and need urgent trauma surgery for fracture management. Another compelling issue is the return towards full trauma operating capacity and elective orthopedic services, avoiding any major risks for the patients and the healthcare worker team, once the peak of COVID-19 pandemic begins to fall [6].

This topic will be a major issue for any orthopedic surgeons and health workers who have to perform surgical procedures on patients with active COVID-19 or recovered patients.

Does any long persistence of SARS-CoV-2 occur in cancellous bone? Should all the operative room (O.R.) team treating healed patients wear protection devices as is done for those infected by COVID-19? How long should we wait before treating a healed patient without preventive measures concerning to O.R. organization and personal protective equipment (PPE)?

The presence or absence of the virus in cancellous bone could help us answer questions about the management of bone donation in COVID-19-recovered patients.

All these questions need to be answered and this paper aims to assess the prevalence of SARS-CoV-2 RNA in cancellous bone samples in patients hospitalized with active or previous COVID-19 disease.

## 2. Materials and Methods

### 2.1. Study Aims

#### 2.1.1. Primary Endpoint

This monocentric prospective observational study aims to assess the prevalence of SARS-CoV-2 RNA in cancellous bone samples in patients with active or previous COVID-19 disease, who have been hospitalized in our hospital Policlinico San Matteo di Pavia (Italy) and have undergone orthopedic surgery. These findings will improve our knowledge on infection from SARS-CoV-2 and its tropism for human tissues.

#### 2.1.2. Secondary Endpoints

We intend to assess the presence level of SARS-CoV-2 RNA in cancellous bone and its association with: (i) disease status (healed or active); (ii) the time since healing in healed COVID-19 patients; and (iii) the viral load and the time since recovery in recovered COVID-19 patients.

### 2.2. Study Design

This is a cross-sectional observational study with both retrospective and prospective collection of data from stored biological samples and clinical charts.

We collected data about 30 consecutive patients from our institution from January 2021 to March 2021. These patients were either affected by acute COVID-19 disease or achieved recovery (negative nasal swab) and required urgent orthopedic surgery. We enrolled them in this clinical trial after informed consent was acquired.

Inclusion criteria were as follows: (i) patients who underwent urgent orthopedic surgery for fractures or other musculoskeletal injury requiring surgery; (ii) patients affected by acute COVID-19 disease or patients who achieved healing (negative nasal swab); (iii) informed consent signed.

Exclusion criteria were as follows: (i) patients younger than 18 years old; (ii) inability to understand the information about the study and to provide informed consent; (iii) incomplete information/data about the previous COVID-19 disease.

Patients’ demographics and surgical data were collected from the databases which included: age, sex, medical history, BMI, date of admission, date of surgery, procedure and injury type, and COVID-19-related symptoms. The American society of anesthesiologists classification (ASA) score for preoperative risk was recorded on the theatre database and used to assign physical status.

COVID-19 infection was diagnosed in respiratory samples by using two different PCR-based assays in the preoperative period. Specific, real-time reverse transcriptase-polymerase chain reaction (RT-PCR) targeting RNA-dependent RNA polymerase (RdRp) and E genes were used to detect the presence of SARS-CoV-2, according to WHO guidelines and Corman et al. protocols [10,11].

Spongy bones from SARS-CoV-2-positive patients were collected in a 3 mL universal transport medium (UTM™, Copan Italia, Brescia, Italy) and stored at −80 °C until analyzed. Extraction of SARS-CoV-2 RNA was performed on inactivated UTM using the Quick-Viral RNA kit (Zymo Research, Irvine, CA, USA) per the manufacturer’s protocol. Viral RNA was eluted with 50 µL of DNase/RNase-free water. The presence of SARS-CoV-2 in the samples was determined using the same assays used for diagnosis [10,11]. A confirmatory assay Cepheid GeneXpert^®^ Xpert^®^ SARS-CoV-2 assay (Cepheid, Sunnyvale, CA, USA) was used to prove positivity obtained previously.

### 2.3. Statistical Aspects

All analyses will be used using Stata 16 (StataCorp, College Station, TX, USA). All tests will be 2-sided. A *p*-value < 0.05 will be statistically significant.

#### 2.3.1. Sample Size

The sample size calculation is based on the desired precision of the prevalence. We plan to be able to enroll about 40 patients in the next 3 months. With this sample size, we will obtain a precision of the estimate of the prevalence at most of 16% (calculated as half the 95% confidence interval (95% CI) in the less-favorable scenario from a mathematical point of view, in the absence of any prior information, of a prevalence of 50%).

#### 2.3.2. Descriptive Statistics

Continuous data will be described with the mean and standard deviation or the median and quartiles, depending on the distribution; categorical variables will be described as counts and percent.

#### 2.3.3. Analysis of the Primary Endpoint

The prevalence will be computed as the ratio of the positive patients to the total enrolled patients, together with its 95% binomial exact confidence interval.

#### 2.3.4. Analysis of the Secondary Endpoints

1. The prevalence for each type of sample will be computed as described above. The comparison will be performed with Fisher’s exact test; the mean difference and 95% CI will be computed.
2. The SARS-CoV-2 load expressed as cycle threshold will be compared between groups with the Mann–Whitney U test.
3. The association of SARS-CoV-2 load and time since healing will be assessed with the Spearman R and its 95% CI.

## 3. Results

Our population consists of: 30 patients, 19 females and 11 males; the mean age was 77 ± 15 years old with a range from 30 to 93 years old; the mean BMI was 25 ± 5.

We divided the orthopedics diagnosis into four main groups: the first one was femoral fracture, the second one was tibial fracture, the third one was humeral fracture and the last one was all the other. Half of the patients (15) were diagnosed with femoral fracture (group 1), three (3) were diagnosed with tibia fracture (group 2), two (2) were diagnosed with humeral fracture (group 3) and ten (10) with other diagnosis (group 4) (Table 1).

We divided patients into three main groups based on different types of surgical operation. The first group included patients undergoing open reduction and internal fixation (ORIF) or close reduction and internal fixation (CRIF). The second group included patients who underwent hip hemiarthroplasty surgery and the last group involved patients undergoing all the other invasive surgery (Table 2). Sixteen (16) patients underwent ORIF or CRIF surgery, six (6) underwent hip hemiarthroplasty, and the remaining eight (8) patients underwent other types of surgery.

Fourteen patients had an ASA score of 3, thirteen had an ASA score of 2, and the remaining three had an ASA score of 1 (Table 3).

Mean days of hospitalization was 6.7 ± 10 ranging from 0 to 39. Eighteen of the thirty patients included in the study had a positive nasopharyngeal swab at the time of surgery. Twelve patients had a negative nasopharyngeal swab at the time of surgery with a mean days since negativization of 138 ± 104 days ranging from 23 to 331 days. Mean days of positivity to the nasal swab were 17 ± 17. As we checked COVID-19-positive patients every 3 days by means of nasal swab, all the patients considered positive in the O.R. had a positive nasal swab after less than 72 h before surgery. Half of the patients (15) had radiological signs of COVID-19 at the chest X-ray, executed right after the positive nasal swab, and nine (9) of them had bilateral signs (Table 4).

We collected a number of COVID-19-related signs and symptoms, including fever, cough, fatigue, sore throat, dyspnea, spO_2_, chest pain, nasal congestion, headache, dizziness, diarrhea, and abdominal pain. All the patients had one or more of these symptoms during their COVID-19 infection. None of them had COVID-19-specific therapy or were vaccinated.

Twenty-nine out of thirty (96.7%) samples were negative for the presence of SARS-CoV-2 RNA. In one sample, low SARS-CoV-2 load (Cycle threshold (Ct) 36.6) was detected but not confirmed using an additional confirmatory assay.

## 4. Discussion

The new coronavirus pandemic severely affected health care systems worldwide. All surgical specialties had to adapt to different innovations, and changes have been introduced with regard to surgical and outpatient activities.

The necessity of placing more attention and resources on the clinical management of COVID-19 patients has caused these changes. The introduction of some new features was necessary to limit the transmission of the virus and thus the infection in health care personnel [12]. In fact, many health care workers treating COVID-19 patients became infected and subsequently died.

It is known that the virus is present in the blood. The current attitude is to exclude blood donors in cases of COVID-19 positivity. Despite this, transmission of infection has never been reported from donor to recipient either in transfusion of blood products or cellular therapies [13].

According to Xia et al., the presence of the virus in tears and conjunctival secretions has only been demonstrated in SARS-CoV-2-infected patients with conjunctivitis, whereas in SARS-CoV-2 patients without conjunctivitis, the presence of the virus was not demonstrated on RT-PCR analysis. Therefore, it has been proved that tears and conjunctival secretions are not a route of virus transmission from patients SARS-CoV-2 who are not suffering from conjunctivitis [14].

Grassi et al. searched for viral RNA within synovial fluid and bone tissue samples taken post-mortem in patients who died of SARS-CoV-2 respiratory complications. Again, the results were negative [15]. To the best of our knowledge, this study is the first one searching for viral nucleic acid in vivo within bone tissue samples.

It is well-known how the virus is transmitted directly. Less known are the capabilities of indirect transmission.

During orthopedic surgery, many power instruments, such as bone saws, drills and reamers are used, and they are known to be producers of blood aerosols. The theory has been therefore postulated that virus transmission to health care workers may occur through these aerosols. Consequently, some authors have defined a series of preventive measures to be taken in case of COVID-19-positive patients undergoing orthopedic surgery [16]. For example, patients requiring skeletal traction, a hand drill can be used, as well as cutting bone with an osteotome, rather than an oscillating saw blade. This could help reduce aerosol production. Another case is hip replacement surgery, where it is preferable to use spoons to prepare the femur, keep the femoral canal dry with gauze and use saline solution in a syringe, instead of pulse lavage to prevent splash of particles of blood [16].

Personal protection equipment (PPE), such as FFP2 masks, face shields and goggles, are commonly used during surgery of COVID-19-active patients to protect the surgeon. The same equipment is used during anesthesiologic procedures and for O.R. cleaning [17]. Orthopedic surgeons and O.R. personnel showed concern about the lack of comparative efficacy, scientific evidence, compliance, shortage of materials, and the side effects of PPE usage. It is known that PPE can negatively affect surgical performance due to the limitation of vision and communication, discomfort, and fatigue. For this reason, we think that PPE should be used only in cases of necessity [18].

The objective of our study was to understand whether or not there was viral RNA shedding with aerosol, posing a risk to health care providers during orthopedic surgical procedures on positive or previously infected patients.

After informed consent was signed, we obtained cortical spongiosa bone tissue samples from the patients during surgery for standard RT-PCR assay. Almost all samples were negative, and the only positivity was not reconfirmed with an additional assay.

Due to its specificity and sensitivity, RT-PCR is a simple, convenient, and efficient technique to search for viral nucleic acid in the samples. Moreover, it is the gold standard for diagnosis. On the other hand, Lin et al. identified in their review several false positives and negatives due to contamination and damage of samples [19].

These results are obtained either among COVID-19-positive patients during surgery or healed COVID-19 patients. We did not find any association between the disease status (healed or active) and the prevalence of positive RT-PCR. We did not find any association either between the time since healing and the prevalence of SARS-CoV-2 positivity or level of SARS-CoV-2 load. In any case, we did not detect the presence of the virus in the bone samples, indicating that it is not a common way of virus transmission.

Considering these findings, we think that the use of PPE to only protect from aerosols produced during surgery is not strictly necessary in both active and healed patients. This does not mean that the use of PPE should not be employed by surgeon and the O.R. personnel to protect themselves from aerosols produced from the respiratory tract.

Another challenge created by the COVID-19 pandemic is the management of the tissue bank, primarily due to the possibility of human tissue contamination or the risk of disease transmission following transplantation. Therefore, tissue banks all over the world established strict preventive measures, or in some cases stopped collecting samples. On the other hand, at the moment, no documented reports have proved any COVID-19 infection following tissue transplantation [20,21]. We think that our results could represent a valid basis for further studies related to the possibility of bone donation in patients who suffered and healed from COVID-19.

The main limitation of the study is the small size of the sample due to the availability of resources. In order to improve the study reliability, it should be implemented with further cases in future studies.

## 5. Conclusions

Our study demonstrates the absence of the viral genome within the analyzed cancellous bone. This leads us to assume that SARS-CoV-2 has no direct bone affinity. We think that the aerosol generated during orthopedic surgery, in patients with active COVID-19 or healed, does not represent a risk of infection for the surgeons and the O.R. personnel. Therefore, PPE should not be necessary for this part of the surgery only.

Despite this, normal activities in the operating room with COVID-19-active patients, such as intubation, moving the patient to the operating table, etc., can promote the dispersion of viral particles into the environment. Consequently, this may cause the infection of health care personnel. It is therefore imperative to take preventive measures in the operating room when performing surgery on a COVID-19-active patient, especially through the use of PPE, in order to prevent virus transmission.

The small number of patients is the main limitation of the study, and for this reason further studies are needed to better understand the exact natural history of the disease.

We think that our results could represent an important basis for further studies related to this topic.

## Figures and Tables

**Table 1:** Diagnosis of fracture.

Diagnosis	N° of Patients (30)
Femoral fracture (group 1)	15
Tibia fracture (group 2)	3
Humeral fracture (group 3)	2
Other diagnosis (group 4)	10

**Table 2:** Type of surgical operation.

Surgical Operation	N° of Patients (30)
ORIF or CRIF	16
Hip hemiarthroplasty	6
All the other	8

**Table 3:** ASA risk score.

ASA Score	N° of Patients (30)
ASA 1	3
ASA 2	13
ASA 3	14

**Table 4:** Signs of COVID-19 infection in chest X-rays.

X-rays Signs of COVID-19 Infection	N° Patients (30)	Bilateral Signs
Yes	15	9
No	15	

## Data Availability

The data presented in this study are available on request from the corresponding author. The data are not publicly available due to privacy restrictions.
